# Heterotrophic bacteria in drinking water: evaluating antibiotic resistance and the presence of virulence genes

**DOI:** 10.1128/spectrum.03359-23

**Published:** 2024-01-11

**Authors:** Lesego G. Molale-Tom, Oluwaseyi S. Olanrewaju, Rinaldo K. Kritzinger, Justine Fri, Cornelius C. Bezuidenhout

**Affiliations:** 1 Unit for Environmental Sciences and Management, North-West University, Potchefstroom, South Africa; 2 Antimicrobial Resistance and Phage Bio-Control Research Laboratory, Department of Microbiology, Faculty of Natural and Agricultural Sciences, North-West University, Mmabatho, South Africa; University of Maryland Eastern Shore, Princess Anne, Maryland, USA

**Keywords:** antibiotic resistance, antibiotic risk assessment, heterotrophic plate count bacteria, pathogenic potential, safe drinking water, whole genome sequence analysis

## Abstract

Heterotrophic bacteria, impacting those with infections or compromised immunity, pose heightened health risks when resistant to antibiotics. This study investigates heterotrophic plate count bacteria in water from North West-C (NWC) and North West-G (NWG) facilities, revealing prevalent β-hemolysis (NWC 82.5%, NWG 86.7%), enzyme production (98%), and antibiotic resistance, especially in NWC. NWG exhibits variations in hemolysin (*P* = 0.013), lipase (*P* = 0.009), and DNase activity (*P* = 0.006). Antibiotics, including ciprofloxacin, persist throughout treatment, with high resistance to β-lactams and trimethoprim (47%–100%), predominantly in NWC. Multiple antibiotic resistance index indicates that 90% of values exceed 0.20, signifying isolates from high antibiotic usage sources. Whole genome sequencing reveals diverse antibiotic resistance genes in heterotrophic strains, emphasizing their prevalence and health risks in water.

**IMPORTANCE:**

This study’s findings are a stark reminder of a significant health concern: our water sources harbor antibiotic-resistant heterotrophic bacteria, which can potentially cause illness, especially in individuals with weakened immune systems or underlying infections. Antibiotic resistance among these bacteria is deeply concerning, as it threatens the effectiveness of antibiotics, critical for treating various infections. Moreover, detecting virulence factors in a notable proportion of these bacteria highlights their elevated risk to public health. This research underscores the immediate need for enhanced water treatment processes, rigorous water quality monitoring, and the development of strategies to combat antibiotic resistance in the environment. Safeguarding the safety of our drinking water is imperative to protect public health and mitigate the spread of antibiotic-resistant infections, making these findings a compelling call to action for policymakers and public health authorities alike.

## INTRODUCTION

Access to safe drinking water is a fundamental human right and should not pose any health hazards when consumed (1). One way to assess the microbiological quality of drinking water is by measuring the amount of heterotrophic bacteria present. Heterotrophic bacteria can be found in various environments, including water sources such as drinking water, ballast water, and seawater (2, 3). Heterotrophic plate count (HPC) bacteria are commonly used to indicate water quality and the effectiveness of water treatment processes (4). The HPC method involves culturing bacteria on agar plates and counting the number of colonies that form, which estimates the total number of viable bacteria present in a sample. HPC measurements are not used to assess the specific health risks associated with individual bacterial strains but rather to monitor changes in the microbial quality of water and to ensure that water treatment processes are effective in reducing bacterial contamination (4, 5). The South African National Standards (SANS 2015:241) specify that good-quality drinking water should have HPC counts below 1,000 CFU/mL to prevent an ideal environment for bacterial growth. Although limited research has been conducted on the effects of heterotrophic bacteria on human health, previous studies suggest that even at low and acceptable levels, these bacteria may have pathogenic effects, particularly in individuals with compromised immune systems, such as infants, people with underlying infections like diabetes or HIV/AIDS, and the elderly (5). Therefore, it is a matter of concern, especially in a country like South Africa, where the prevalence of HIV/AIDS is high, with approximately 8.2 million people living with the disease as of 2021 (available from StatsSA: https://www.statssa.gov.za/publications/P0302/P03022021.pdf). Studies have identified opportunistic pathogens among naturally occurring heterotrophic bacteria, including species such as *Aeromonas* spp., *Acinetobacter* spp., *Bacillus* spp., *Klebsiella* spp., *Moraxella* spp., *Flavobacterium* spp., *Mycobacteria* spp., *Pseudomonas* spp., *Serratia* spp., and *Xanthomonas* spp. (6, 7).

Generally, the drinking source water always contains bacteria that are primarily part of the natural composition of water environments and other contaminating bacteria resulting from anthropogenic activities (8). Source waters have also been contaminated with antibiotic residue, antibiotic-resistant bacteria (ARB), and antibiotic resistance genes (ARGs), which have been reported to be widespread in aquatic environments due to the extensive global and inappropriate use of antibiotics in agriculture, veterinary, and human medicine (9). These contaminants may find their way through runoffs into surface waters (10). ARB and ARGs may also find themselves in surface waters through improperly treated animal and fecal waste (11), which is evident by studies that reported the presence of these contaminants in treated effluents (12). These sources serve as reservoirs contributing to the growth and further spread of antimicrobial-resistant microorganisms in the environment. In the past, concerns about water’s microbial quality have centered on pathogens’ occurrence in drinking water distribution systems, and effective disinfection has been used to inactivate waterborne pathogens (13). Although there is a significantly low concentration of microorganisms in treated water, sterilization is impractical. Therefore, the developing problem is the presence of trace amounts of contaminating ARB and antibiotics from source to finished drinking water that significantly negatively impact public health (11). Sullivan et al. (14) conducted comparable research in which the concentration of antibiotic-resistant bacteria and antibiotic-resistant genes was examined before and after chlorination. The proportion of antibiotic-resistant microbes and antibiotic-resistant genes was higher after chlorination. This also demonstrates that disinfection procedures do not always decrease bacteria but can also select antibiotic-resistant bacteria and their related genes. Therefore, source waters supplying drinking water treatment facilities shown to contain antibiotic residues, ARB, and ARGs may bypass treatment processes and enter drinking water distribution systems (11, 15). These genes may be contained in potentially pathogenic heterotrophic bacteria that can resist currently used antibiotic therapies or, through horizontal gene transfer, be a source of resistance traits to susceptible pathogenic bacteria.

The practice of depending on abiotic parameters in addition to bacterial enumeration only to indicate water quality, therefore, overlooks the full potential biological impact of previously considered health-risk-free heterotrophic bacteria on humans. Considering the potential pathogenicity, antibiotic resistance, and virulence profiles of these heterotrophic strains would indicate the potential risk of heterotrophic bacteria on human health. Recent advances in molecular techniques have been applauded for their high rapidity, sensitivity, and specificity and enable the detection and quantification of even a very low number of targets in a matter of hours as well as reveal an in-depth characterization of the microbial cells (16). Whole genome sequencing (WGS) can provide the genetic profile of a sample and its antimicrobial resistance (AMR) characterization, revealing its pathogenicity. Genome mining approaches have been used in various studies to identify pathogenicity and virulence factors present in bacterial isolates (17, 18), showing the significance and importance of this approach in safe drinking water.

Hence, this study aims to identify the pathogenic properties and antibiotic resistance patterns of heterotrophic bacteria isolated from raw, treated, and drinking water from two drinking water production facilities in South Africa. One with minimal treatment processes North West-C (NWC) and a second with conventional treatment processes North West-G (NWG) were investigated using phenotypic and whole genome sequence analysis to provide valuable insights for water treatment processes and public health management.

## MATERIALS AND METHODS

### Bacterial isolates and description of treatment plants

This study used a total of 127 confirmed heterotrophic bacterial isolates. These isolates were recovered from two drinking water production facilities, NWC and NWG, in the North West Province of South Africa. The isolates were obtained from samples collected at different stages of the water treatment process, including raw water, finished (treated) water, and distribution points (taps).

NWC is situated in a local municipality with approximately 56,702 inhabitants. It has limited urban development and fewer industries that could pollute the environment. The filtration plant capacity of NWC is 350 m^3^/h, and the source water is obtained from a nearby river, which is considered clean (19). The treatment processes at NWC are elementary: physical separation, sand filtration, and disinfection (chlorination) (20). On the other hand, NWG is a larger plant that purchases raw water from the Department of Water and Sanitation, purifies it, and distributes it to consumers across an area of approximately 900 km^2^ with approximately 148,804 inhabitants. The raw water source for NWG is a river that is extensively utilized and constantly exposed to various pollutants. The treatment processes at NWG include pre-ozonation, coagulation-flocculation, dissolved air flotation, ozonation, sedimentation, filtration, and chlorination.

The heterotrophic bacterial isolates were recovered from water samples collected from NWC and NWG following the DWAF sampling guide (21). The samples were collected in March, May, and August 2016 as well as in May and November 2017 from raw water, treated water, and household taps. A total of 30 samples were collected per site per year. The samples were collected in 1-L sterile Schott bottles and transported under cool conditions to the laboratory for further analysis within 6 hours of collection. The laboratory isolation and identification of the heterotrophic bacterial isolates have been previously described by Kritzinger (22). There were no specific criteria to seek for more resistant isolates. We investigated all isolates and determined their resistance. The antibiotics utilized represent a broad spectrum against Gram-positive and Gram-negative bacteria, which allowed us to determine the sensitivity of the isolates against a wide range of antibiotics.

### Detection of antibiotic residues in water samples

The water samples collected were also subjected to quantification of selected antibiotic residues. Target compounds in the water samples were concentrated 2,000 times by automated solid phase extraction (SPE) using the SPE-DEX system (Horizon Technology, Salem, NH, USA). The resulting eluents were reconstituted in methanol and subjected to UPLC-QTOF for analysis. The following antibiotics were tested: ampicillin, cephalothin, chloramphenicol, ciprofloxacin, erythromycin, kanamycin, neomycin, oxytetracycline, benzyl-penicillin (penicillin G), penicillin, streptomycin, trimethoprim, and sulfamethoxazole.

### Phenotypic pathogenicity assays

The potential pathogenicity of heterotrophic bacteria was assessed by examining isolates for various extracellular enzymes, including hemolytic activity, proteinase, lipase, DNase, and lecithinase. The isolates were cultured at temperatures ranging from 28°C to 30°C, as most heterotrophic isolates did not grow well at 37°C. Incubation times for certain enzyme tests, such as proteinase, lipase, and lecithinase, were adjusted to prevent plate overgrowth.

Pure isolates were sub-cultured on 5% (wt/vol) sheep blood agar and incubated at 28°C to 30°C for 24 hours to determine hemolytic activity. Colonies were then categorized as alpha (α), beta (β), or gamma (γ) hemolytic strains (23). Proteinase production was assessed using brain-heart infusion agar supplemented with 3% skimmed milk. Plates were incubated at 28°C to 30°C for 24 hours, and transparent zones around the colonies were observed as indicators of proteinase activity (24).

For lipase activity determination, isolates were cultured on tryptone soy agar (Merck, Germany) supplemented with 1% Tween-80 (Sigma, Germany). Plates were incubated at 28°C to 30°C for 48 hours, and a turbid halo around the inoculation spot was checked to confirm lipase activity (25). DNase production was assessed using DNase agar (Merck, RSA) supplemented with toluidine blue (Sigma, Germany). Plates were incubated at 28°C to 30°C for 48 hours, and clear zones around the colonies indicated DNase activity (26).

Lecithinase production was determined by culturing isolates on McClung-Toabe egg yolk agar supplemented with 50% egg yolk mix (Merck, RSA). After 24 hours of incubation at 28°C to 30°C, evidence of egg yolk degradation was examined to confirm the lecithinase activity (27).

### Antibiotic susceptibility of HPC bacteria

The antibiotic susceptibility test used the disc diffusion method (28). Pure isolates were subcultured in R2A broth and incubated at room temperature (approximately 25°C) for 5–7 days. A standardized broth culture (100 µL) was spread plated on Mueller-Hinton agar plates, followed by antibiotic discs. Twelve antibiotics (Mast Diagnostics, UK) were tested: ampicillin (10 µg), cephalothin (30 µg), chloramphenicol (30 µg), ciprofloxacin (5 µg), erythromycin (15 µg), kanamycin (30 µg), neomycin (30 µg), oxytetracycline (30 µg), penicillin G (10 units), streptomycin (25 µg), trimethoprim (5 µg), and vancomycin (30 µg).

The plates were incubated at 37°C for 24 hours, and the interpretation of inhibition zones was conducted according to critical values recommendations based on the Clinical and Laboratory Standards Institutes (https://em100.edaptivedocs.net/GetDoc.aspx?doc=CLSI%20M45%20ED3:2016&scope=user). The antibiotic resistance rate was defined as the mean percentage of total isolates resistant to each tested antibiotic at NWC and NWG sampling sites.

Multiple antibiotic resistance (MAR) was defined as resistance to three or more antibiotic classes. MAR values were calculated per sampling site using the MAR index formula: MAR index=a÷(b×c) per sample, where *a* represents the aggregate antibiotic resistance of all isolates, *b* is the number of antibiotics tested, and *c* is the number of isolates in the sample. Intermediate resistant (I) and resistant (R) isolates were included in the calculation as resistant to antibiotics.

### Statistical analysis

A chi-squared (χ^2^) test and, where necessary, the Fisher exact test were used to determine if there were significant changes in the extracellular enzyme activity between the raw and drinking water isolates for each purification plant. The antibiotic resistance rates were defined as the mean percentage of total isolates resistant to the selected antibiotics tested for the sampling sites. The χ^2^ test was also used to determine any associations between resistant isolates in the raw and treated isolates and between resistant strains isolated from treated and distributions for each study site. Statistical significance was set at *P* < 0.05.

### Whole genome sequencing and bioinformatic analysis

WGS was performed on a subset of multidrug-resistant heterotrophic isolates, with two each obtained from raw and distribution water. DNA from pure isolates was isolated, quantified as previously described, and used for 250-bp paired-end sequencing on an Illumina MiSeq sequencer.

The raw reads obtained underwent quality-based trimming and filtering using Trimmomatic (v0.36) (29). Fragments with low-quality values from each end, adaptors, and short reads (less than 50 nucleotides) were removed from further analysis. The read pairs were assembled independently using SPAdes version 3.9.0 (30). Genome annotation was performed using Prokka, a software tool for rapid prokaryotic genome annotation (31).

The annotated file was then subjected to BLAST assessment against the Genome Taxonomy Database using GTDB-Tk version 1.7.0 (32). The genome and its typical features were visualized using the proksee server (33). Genomic islands within the genomes were predicted using IslandViewer 4 server (34).

Antibiotic-resistant genes and virulence genes were identified using DeepARG (35) and the Resistance Gene Identifier (RGI) on the Comprehensive Antibiotic Resistance Database (CARD) (36). The RGI utilizes the contigs file with the parameters “Perfect and strict hits only” and “High quality/coverage,” which increases more specificity while DeepARG models exhibit good precision and greater total recall compared to standard best-hit techniques, resulting in continuously reduced false-negative rates. Phage annotation was performed using the PHAge Search Tool with Enhanced Sequence Translation (PHASTEST) web server (37). The antibiotic resistance genes and virulence genes were viewed using circos (38).

### Phylogenomic and comparative genomic analysis

A phylogenetic tree was generated utilizing the average nucleotide identity (ANI) for analysis. The Orthologous Average Nucleotide Identity Tool (OAT) v0.93.1 (39) was utilized to determine the overall similarity among the whole genome sequences. The genomes and their respective species were subjected to syntenic analysis using the Mauve Genome Alignment (version 2.3.9) (40), with the progressive Mauve algorithm and default parameters.

## RESULTS

### Antibiotics in water sample

Ciprofloxacin was detected in all tested samples (Table 1). Penicillin was found in all samples from NWC, while neomycin was detected in treated and distribution water from NWC. Benzyl-penicillin (penicillin G) was found in raw and distribution water from NWG, while streptomycin was detected in raw and treated water from NWG (Table 1). The presence of antibiotics such as ciprofloxacin, neomycin, benzyl-penicillin (penicillin G), and streptomycin in the water samples suggests contamination with these antibiotics. This contamination raises concerns about the potential impact on human health and the environment. Hence, continuous monitoring and appropriate water treatment processes in these plants are necessary to ensure the removal or reduction of antibiotic residues in distributed drinking water, thereby minimizing the potential associated risks.

**TABLE 1:** Summary of antibiotics detected in the drinking water treatment stages

Isolate source	Antibiotic(s)
NWC raw	Ciprofloxacin, penicillin
NWC treated	Ciprofloxacin, penicillin, neomycin
NWC distribution	Ciprofloxacin, penicillin, neomycin
NWG raw	Ciprofloxacin, benzyl-penicillin, streptomycin
NWG treated	Ciprofloxacin
NWG distribution	Ciprofloxacin, benzyl-penicillin, streptomycin

### Potential pathogenicity of the isolates

Overall, a significant proportion of the isolates showed potential pathogenic characteristics (Table 2). Remarkably, 87% of the NWC source water isolates and 82.5% of the NWC final water isolates tested positive for hemolysin. Among these, only one isolate from the raw water and one isolate from the drinking water exhibited α-hemolytic activity, while the remaining isolates showed β-hemolytic activity. A majority of the source water strains (76.5%) and drinking water strains (86.7%) from NWG also displayed hemolytic activity. Similarly, a high percentage (85%–97%) of isolates from both plants were found to be producers of proteinase and lecithinase. However, lipase production was observed in 38.5% of NWG isolates, which decreased to 19.2% in drinking water isolates. Approximately 40%–50.5% of the isolates in the source water and final water from both production facilities exhibited DNase activity. This result raises concerns about the potential health risk associated with drinking water from both facilities.

**TABLE 2:** Number and percentage of isolates showing pathogenic activity

Isolate source	Number tested (n*, *n*)^*a*^	Hemolysis	Proteinase	Lecithinase	Lipase	DNase
NWC raw	23*, 20	20 (87)	17 (85)	18 (90)	9 (45)	8 (40)
NWC drinking	40*, 33	33 (82.5)	31 (93.9)	32 (97)	13 (39.4)	17 (51.5)
NWG raw	34*, 26	26 (76.5)	25 (96.2)	25 (96.2)	10 (38.5)	11 (42.3)
NWG drinking	30*, 26	26 (86.7)	25 (96.2)	25 (96.2)	5 (19.2)	13 (50)

^*a*^ n* = number of isolates tested for hemolysin activity; *n* = number of isolates tested for other enzymes.

At the NWC facility, the proportion of enzyme producers did not show a significant association (*P* > 0.05) with the isolate sources, whether they were sourced from raw water or drinking water (Table 3). In contrast, significant relationships (*P* < 0.05) were observed between extracellular enzyme production and isolate source at the NWG facility. This indicates that the source of the isolates (raw or drinking water) had a statistically significant impact on the production of extracellular enzymes among the isolates at NWG.

**TABLE 3:** Association between the isolate source (raw or drinking) and extracellular enzyme production

		Hemolysin	Proteinase	Lecithinase	Lipase	DNase
NWC	*P*-values	0.734	0.354	0.549	0.688	0.416
NWG	*P*-values	0.013^*a*^	0.057	0.057	0.009^*a*^	0.006^*a*^

^*a*^ Indicative of significance (*P* < 0.05).

Among the 127 isolates tested for extracellular enzyme production, three isolates (two *Pseudomonas protegens* and one *Sphingomonas kaistensis*) were positive for two enzyme tests, namely, hemolysin and lipase. The majority of the isolates (97.6%, *n* = 124) were positive for three or more of the tested enzymes. Notably, nine isolates (7.1%) tested positive for all extracellular enzymes. These included five isolates from NWC drinking water (*Bacillus* sp., *B. paramycoides*, *B. sonorensis*, *Arthrobacter* sp., and *Arthrobacter oryzae*), three isolates from NWG raw water (*Bacillus licheniformis*, *Shewanella* sp., and *Deefgea rivuli*), and one isolate (*Aeromonas salmonicida*) from NWG drinking water.

### Antibiotic susceptibility

Table 4 displays the average proportion of bacterial isolates resistant to each antibiotic for NWC and NWG. The results indicated high average resistance against β-lactams (ampicillin, cephalothin, and penicillin) and trimethoprim, ranging from 47% to 100% for both drinking water treatment systems.

**TABLE 4:** The average percentages of isolates resistant to the selected antibiotics during all the sampling sessions at NWC and NWG^*a*^

Plant	Site	*n*	AMP%	KF%	P%	TMP%	E%	VA%	C%	CIP%	K%	NE%	OT%	S%
NWC	R	47	63 ± 23.24	66 ± 182.82	67 ± 31.18	83 ± 13.75	40 ± 24.44	42 ± 35.36	26 ± 30.94	17 ± 16.46	17 ± 9.76	29 ± 27.60	22 ± 25.42	23 ± 27.37
	T	23	60 ± 43.46	61 ± 38.90	100 ± 0.00	73 ± 43.46	13 ± 21.65	25 ± 50.00	10 ± 22.36	20 ± 44.72	7 ± 14.91	11 ± 23.85	0 ± 0.00	0 ± 0.00
	D	42	64 ± 13.72	56 ± 14.01	76 ± 16.58	75 ± 23.29	24 ± 20.25	30 ± 28.28	8 ± 12.44	14 ± 17.56	22 ± 21.03	18 ± 21.53	31 ± 24.49	10 ± 22.36
NWG	R	66	63 ± 32.12	56 ± 29.48	59 ± 36.12	62 ± 25.38	38 ± 21.72	10 ± 22.36	12 ± 7.64	4 ± 5.48	9 ± 12.45	8 ± 10.31	7 ± 8.21	9 ± 9.36
	T	8	47 ± 41.11	47 ± 41.11	58 ± 52.04	56 ± 50.92	11 ± 19.24	0 ± 0.00	11 ± 19.24	0 ± 0.00	22 ± 38.49	11 ± 19.24	0 ± 0.00	11 ± 19.24
	D	50	72 ± 25.55	67 ± 22.55	61 ± 47.28	72 ± 21.73	50 ± 38.72	23 ± 43.46	18 ± 20.54	6 ± 8.52	23 ± 35.14	12 ± 26.83	7 ± 10.11	22 ± 25.49

^*a*^ AMP = ampicillin (10 µg); KF = cephalothin (30 µg); C = chloramphenicol (30 µg); CIP = ciprofloxacin (5 µg); E = erythromycin (15 µg); K = kanamycin (30 µg); NE = neomycin (30 µg); OT = oxytetracycline (30 µg); P = penicillin-G (10 units); S = streptomycin (25 µg); TMP = trimethoprim (5 µg); VA = vancomycin (30 µg); R = raw; T = treated; D = distribution; *n* = number of isolates.

At NWC, there was a high prevalence of resistance to ampicillin, cephalothin, penicillin G, and trimethoprim throughout the water treatment continuum, ranging from 63% to 83% in raw water, 60% to 100% in treated water, and 64% to 76% in drinking water at distribution taps. Notably, penicillin resistance showed an average of 67% in raw water, which increased to 100% in treated/finished water and then decreased to 76% in distribution water. Trimethoprim’s average resistance was 83% in raw water, decreasing to 73% in treated water and 76% in distribution water at NWC.

At NWG, the average resistance of raw water isolates was 63% for ampicillin and 56% for cephalothin. In treated water, average resistance was 47% for both ampicillin and cephalothin but increased to 72% and 67%, respectively, in drinking water. The average resistance against penicillin at NWG was lower than at NWC, ranging from 58% to 61% in raw and drinking water. Similarly, resistance to trimethoprim was lower at NWG compared to NWC, with percentages of 62% in raw water, 56% in treated water, and 72% in distribution water.

A general trend observed was increased average resistance from raw to drinking water for NWC and NWG. Apart from β-lactam antibiotics and trimethoprim, resistance to other antibiotics ranged from 0% to 42%, with higher resistance associated with NWC than NWG.

### Multiple antibiotic resistance

Three of the nine isolates recovered from NWC drinking water that secreted all tested enzymes showed multiple antibiotic resistance patterns. *Bacillus* sp. exhibited resistance against ampicillin, cephalothin, penicillin, trimethoprim, and vancomycin. *B. paramycoides* displayed resistance to ampicillin, cephalothin, penicillin, trimethoprim, erythromycin, and kanamycin. *B. sororensis* demonstrated resistance to ampicillin, cephalothin, penicillin, trimethoprim, kanamycin, and oxytetracycline.

Table 5 presents the multiple antibiotic resistance index (MARI) calculated for NWC and NWG across all sampling sessions. At NWC, the MARI values ranged from 0.29 to 0.47 for raw water, 0.12 to 0.38 for treated water, and 0.24 to 0.38 for distribution water isolates. Similarly, the MARI values for raw, treated, and distributed water isolates from NWG were recorded as 0.15 to 0.35, 0.08 to 0.35, and 0.13 to 0.56, respectively.

**TABLE 5:** MARI of heterotrophic bacterial strains

	Raw		Treated		Distribution	
Sampling date	NWC	NWG	NWC	NWG	NWC	NWG
March 2016	0.32	0.28	0.25	–^*a*^	0.44	0.51
May 2016	0.29	0.15	0.31	–^*a*^	0.24	0.13
August 2016	0.47	0.35	0.37	0.27	0.38	0.28
May 2017	0.39	0.28	0.38	0.35	0.28	0.56
November 2017	0.46	0.35	0.12	0.08	0.35	0.35

^*a*^ –, no result was obtained.

The mean MARI values were higher in raw and treated water at NWC, consistent with the higher antibiotic resistance observed previously in NWC compared to NWG. Notably, 90% of the calculated MARI values exceeded 0.20, indicating high-risk sources of isolates, particularly in areas with high antibiotic usage.

### Genome properties and molecular identification

**Fig 1:**
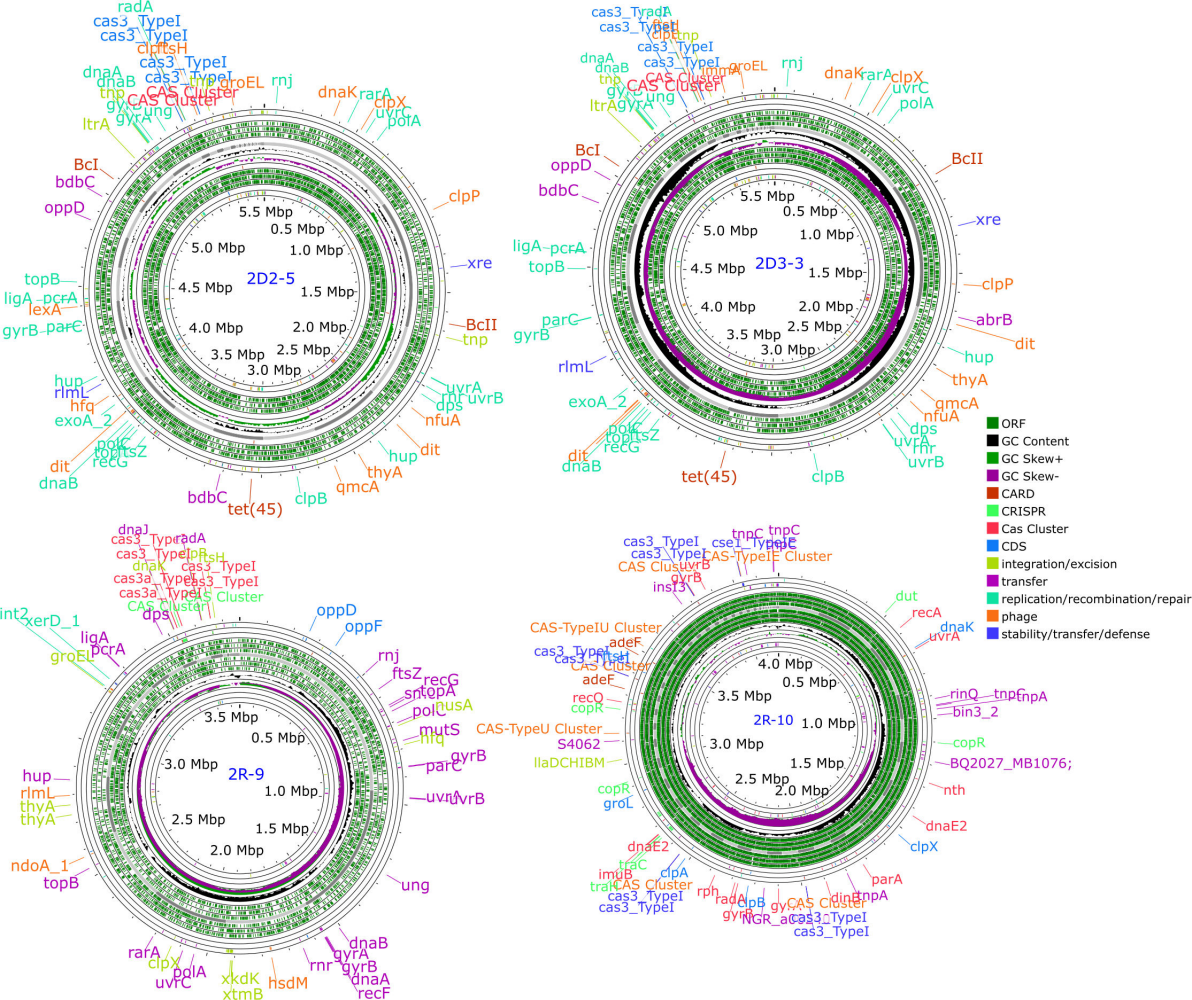
The circular annotated genome view of the strains.

Four HPC isolates (two each from raw and distributed water) were selected for whole genome sequencing and mining. Three were *Bacillus* spp. and one *Sphingomonas* sp. and were analyzed by WGS revealing total genome sizes varying between 3.6 Mbp and 6.5 Mbp (Fig. 1).

**Fig 2:**
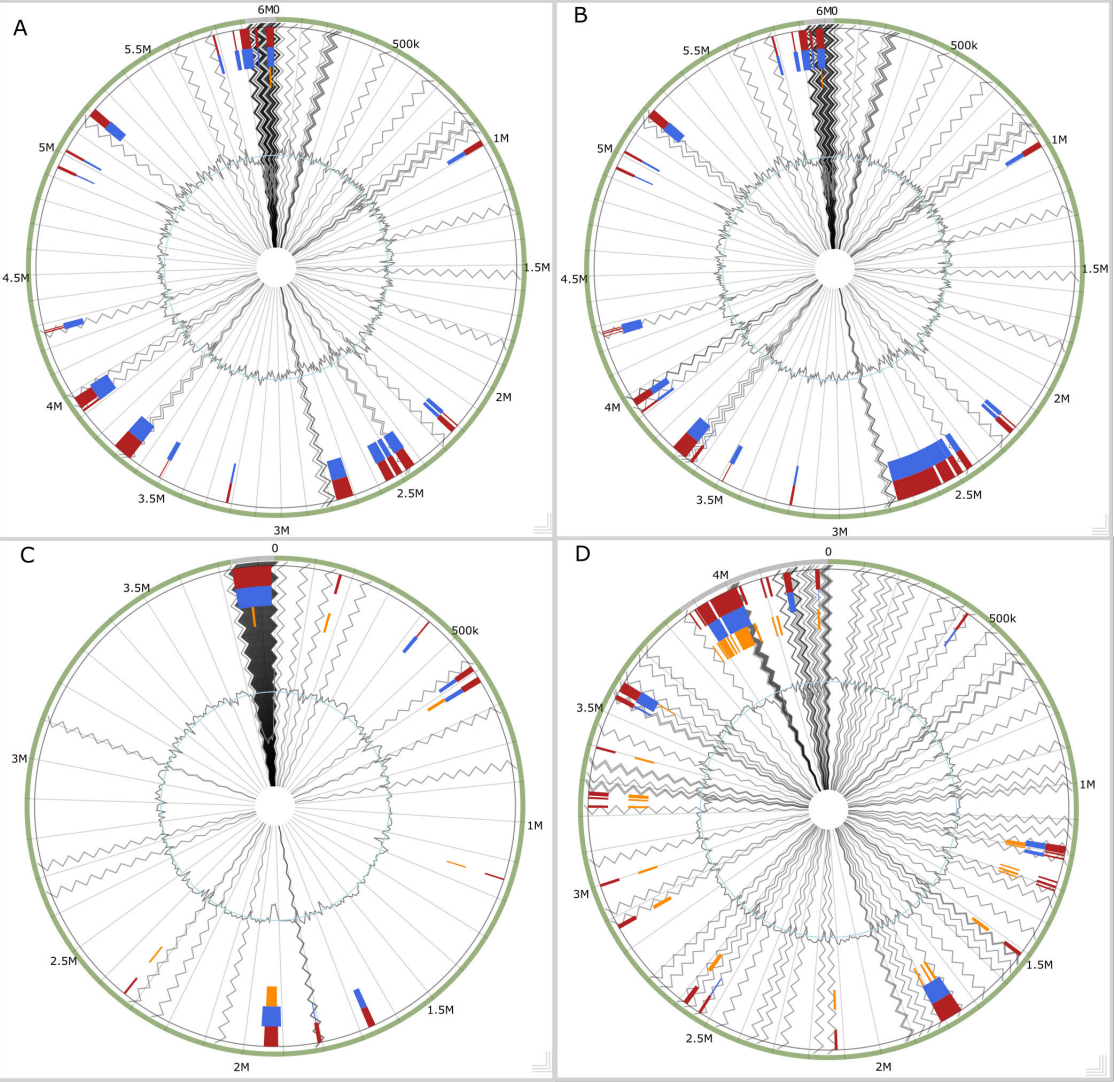
Circular plot of the GIs identified in the chromosomes. (**A**) 2D2-5, (**B**) 2D3-3, (**C**) 2R-9, and (**D**) 2R-10. The orange bars represent the predicted GIs identified by SIGI-HMM, the blue bars represent the analysis by IslandPath-DIMOB, and the red boxes represent the integrated search results.

Genome islands (GIs) often provide adaptive traits that enhance bacterial fitness within a niche (41). Some genome islands were identified in the genomes (Fig. 2). The functions of some genomic islands are for stress resistance, volatile organic compound (VOC) production, and antimicrobial resistance. However, many of the genomic island functions are unknown. These results suggest that the genes from genomic islands probably had a horizontal origin from other bacteria in different niches via different methods. Some of the identified island-encoded genes include collagen adhesin gene, exonuclease *SbcC* gene, resolvase gene, phenazine biosynthesis protein *PhzF* like β-lactamase class C-like and penicillin-binding protein superfamily, tetracycline resistance, MFS efflux pump => Tet(45), mobile element proteins, bacitracin efflux ABC transporter, permease protein *BcrAB*, antigen, stage V sporulation protein *SpoVK*, and virulence-associated protein E, among others (Tables S1 to S4).

### Phylogenomics and comparative genomics

**Fig 3:**
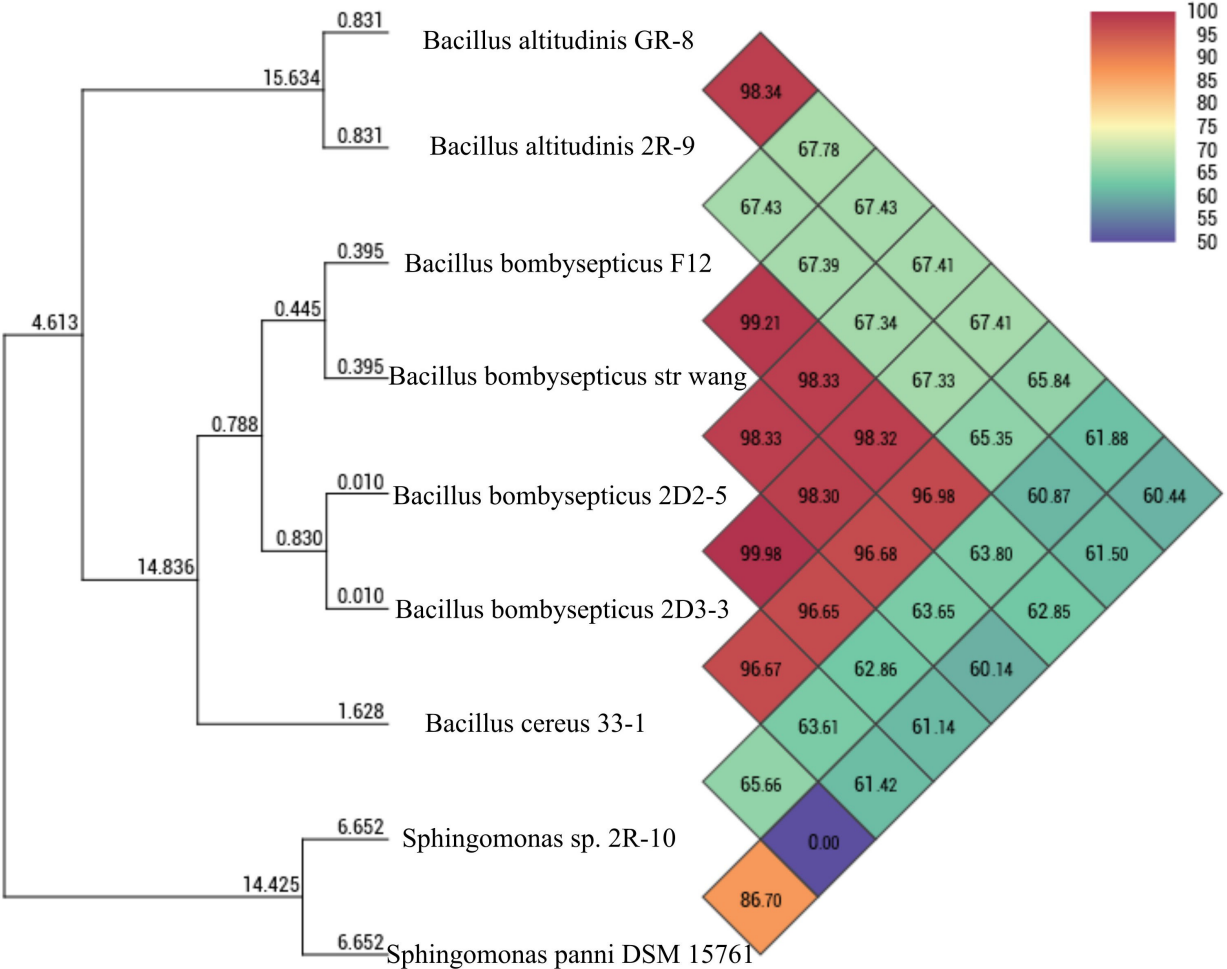
ANI demonstrating nucleotide-level genomic similarity between the coding regions of the isolate genomes. Pairwise comparisons for all represented genomes were computed using the OAT Program.

Taxonomic and functional research on microorganisms has become increasingly reliant on genome-based data and techniques (42). Genome-based identification by the Genome Taxonomy Database (GTDB) identified 2D2-5 and 2D3-3 as *Bacillus bombysepticus* species, 2R-9 as *Bacillus altitudinis*, and 2R-10 as a *Sphingomonas* species (Table S5). Furthermore, the genomes were subjected ANI analysis, and the results of the ANI genome-based phylogenetic analysis are presented in Fig. 3. DNA-DNA hybridization (DDH) and ANI have emerged important for prokaryotic species circumscriptions at the genomic level (43). Genome-genome distance calculator (GGDC) which mimics the DDH was used to calculate the genome distances among the species. In contrast to the proposed threshold of 95% for bacterial species delineation (43), the ANI values between the strain 2R-10 and sequenced genome of *Sphingomonas panni* DSM 15761 is 86.70%, while the GGDC is 0.13 (Table S6). On the other hand, 2D2-5 and 2D3-3 had ANI values of 98.33% and 98.30% with sequenced genome of *B. bombysepticus* str. Wang. The ANI result and the phylogenomics further confirm the result obtained from the GTDB analysis. In addition, 2R-10 is likely to be a new species of *Sphingomonas* according to the GTDB version 2.3.0 (Table S5).

**Fig 4:**
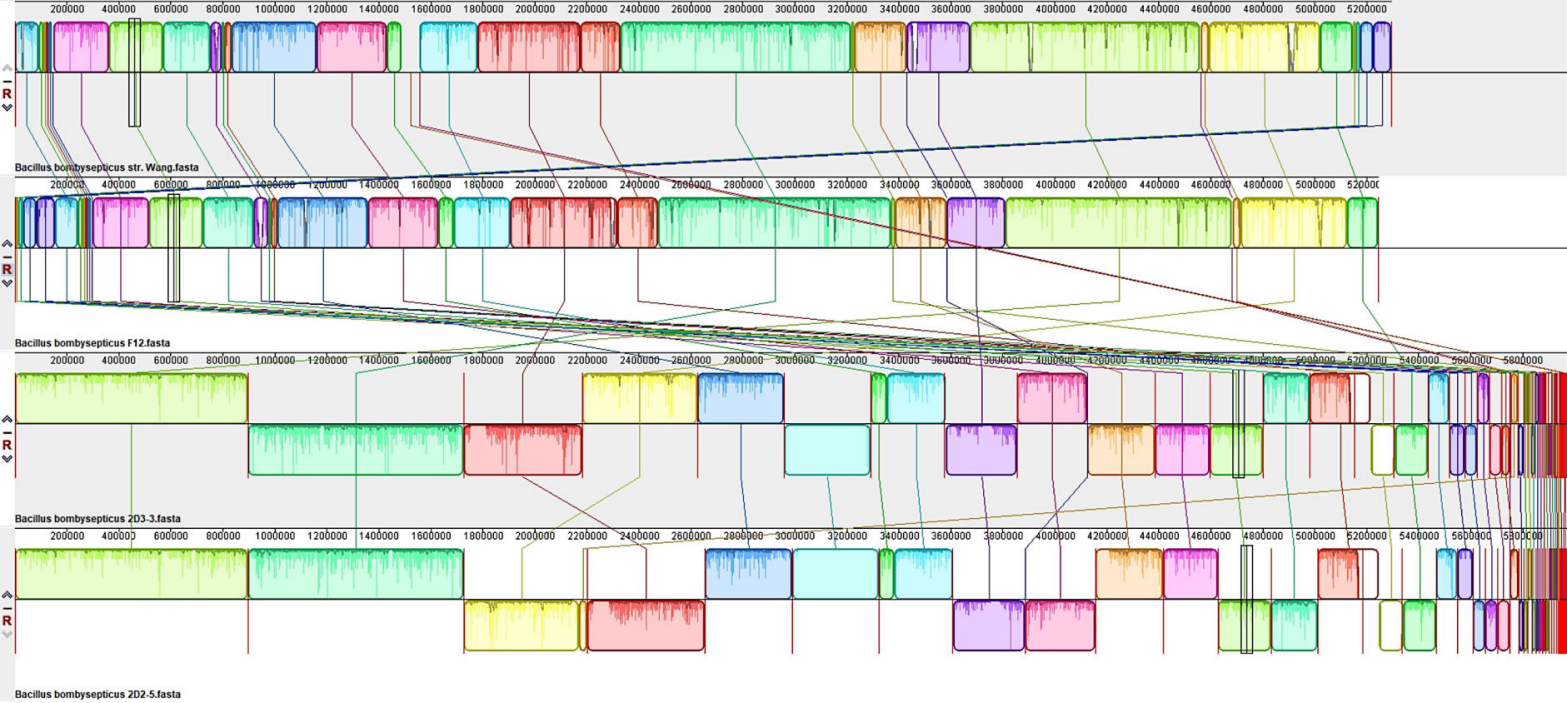
Comparative synteny line plots of the whole genome sequences of *Bacillus bombysepticus* str. Wang, *Bacillus bombysepticus* F12, *Bacillus bombysepticus* 2D3-3, and *Bacillus bombysepticus* 2D2-5.

**Fig 5:**
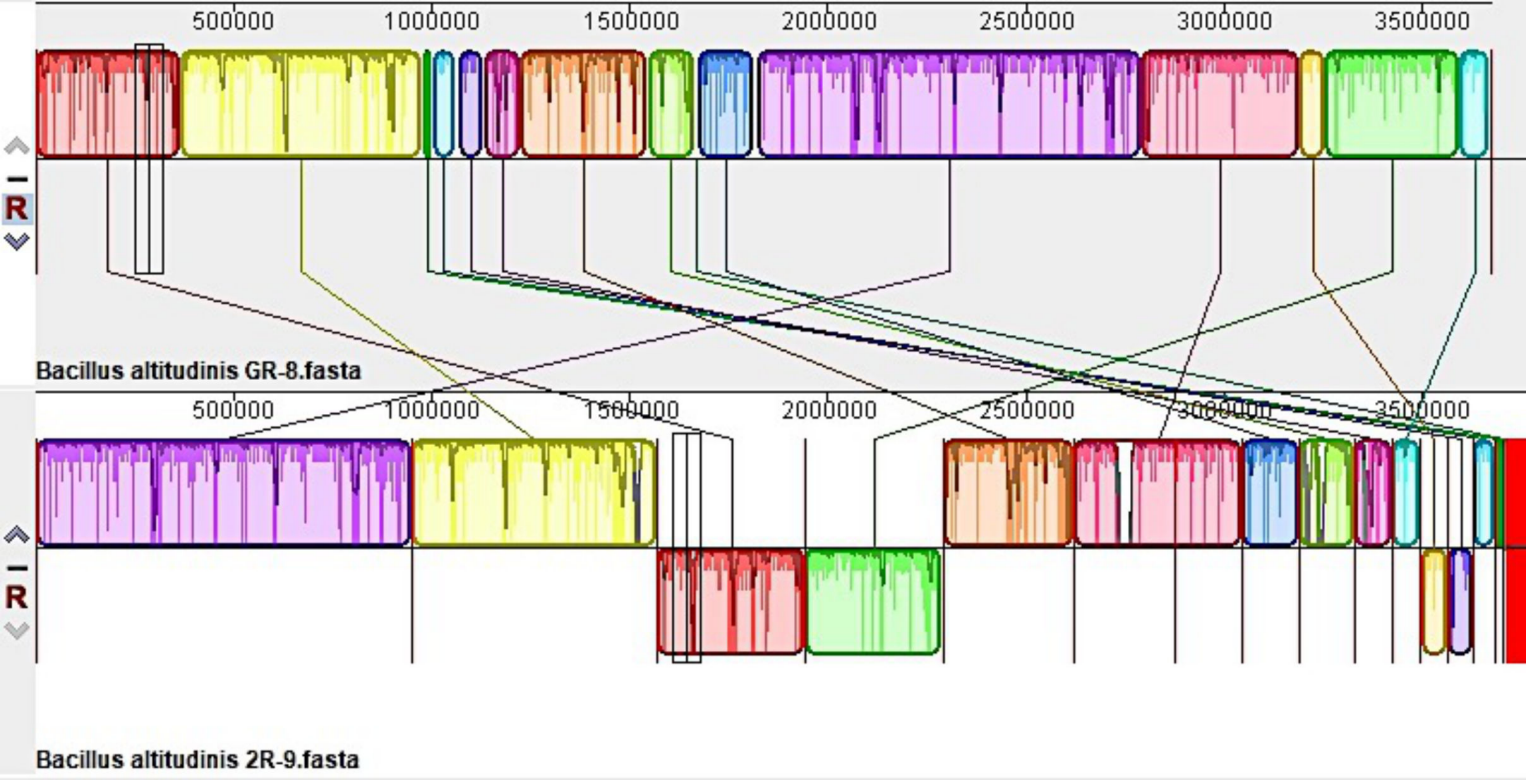
Comparative synteny line plots of the whole genome sequences of *Bacillus altitudinis* GR-8 and *Bacillus altitudinis* 2R-9.

**Fig 6:**
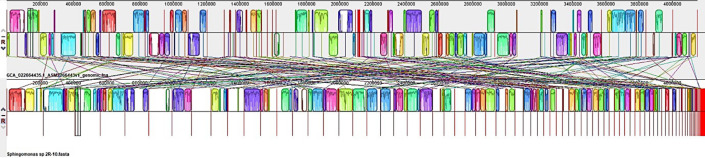
Comparative synteny line plots of the whole genome sequences of *Sphingomonas panni* DSM 15761 and *Sphingomonas* sp. 2R-10.

Figure 4 illustrates the genome structure comparisons between 2D2-5 and 2D3-3 and their closely related species, namely, *B. bombysepticus* str. Wang and *B. bombysepticus* F12. Figure 5 depicts a comparison of genome structure between 2R-9 and its closely related species, *B. altitudinis* GR-8. While Fig. 6 compares genome structure between 2R-10 and *Sphingomonas panni* DSM 15761, the representation of each colored region corresponds to a locally colinear block (LCB), which denotes areas of homologous backbone sequence. The LCBs located below the genome center lines exhibit a reverse complement orientation with respect to the reference genome. The tracing of orthologous LCBs across all genomes is depicted by the lines connecting the genomes. The LCB displays white regions that signify the existence of sequences specific to a particular lineage, whereas red lines denote sequences that are distinct and do not exhibit any resemblance to those found in other organisms. The analysis of gene distribution within genomes has revealed the presence of distinct genes, while the alignment of genomes has indicated significant DNA rearrangement, as evidenced by the blue lines. Such rearrangements have probably been influenced by repeat sequences present within the genome.

Moreover, it can be observed from Fig. 3 that there is a greater similarity in nucleotide levels between 2D2-5 and 2D3-3, thereby substantiating their proximity. The nucleotide-level similarity between the chromosomes of 2D2-5 and 2D3-3, as depicted in Fig. 4, provides additional evidence for the close relationship between these strains within the *Bombysepticus* species, indicating the formation of a sub-clade.

### Presence of phages

**Fig 7:**
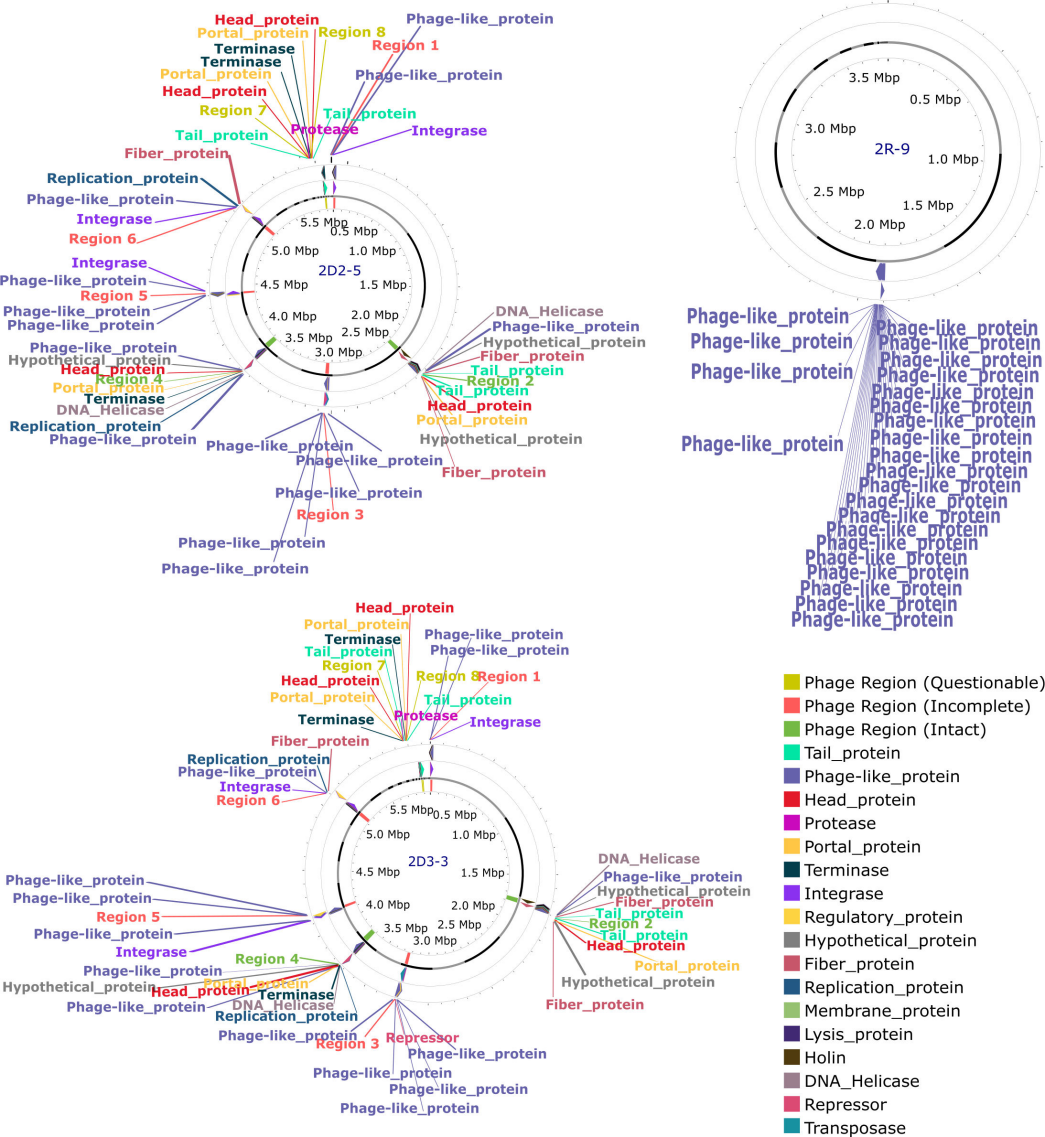
Phage prediction by PHASTEST.

The genomes were analyzed to determine the existence of prophages and plasmids. The findings indicated the absence of phage in the genome of *Sphingomonas* sp. 2R-10. The genomes of *Bacillus bombysepticus* 2D2-5 and 2D3-3 as well as *Bacillus altitudinis* 2R-9 were found to contain eight, eight, and one phage regions, respectively, as illustrated in Fig. 7 and Table 6 and detailed in Tables S7 and S8. Among the identified regions, it was observed that the two strains of *B. bombysepticus* exhibited a total of two intact, four incomplete, and two questionable phage regions each. Conversely, *B. altitudinis* only displayed one questionable phage region that was detected. The genomes did not exhibit any evidence of plasmid presence. Table 6 displays the positions and hits of various coding sequences. Overall, the phage proteins detected include PBSX and DnaB/DnaD.

**TABLE 6:** Phage summary report

Region	Completeness	T. Prot	Region position	Most common phage
*Bacillus bombysepticus* 2D2-5				
NODE_1_length_896591_cov_48.602639	Incomplete	31	229-21688	Bacill_PfEFR_4_NC_048641(8)
NODE_4_length_454455_cov_34.308181	Intact	55	17783-64029	Bacill_phBC6A51_NC_004820(31)
NODE_6_length_333190_cov_50.893525	Incomplete	28	40698-78333	Brevib_Abouo_NC_029029(3)
NODE_8_length_278590_cov_42.650000	Intact	41	135530-190542	Geobac_GBSV1_NC_008376(8)
NODE_10_length_260450_cov_38.865160	Incomplete	23	238294-260258	Bacill_phi4J1_NC_029008(3)
NODE_14_length_171494_cov_30.994795	Incomplete	39	133494-169236	Bacill_phBC6A52_NC_004821(12)
NODE_29_length_11867_cov_43.983220	Questionable	13	2280-11693	Lister_2389_NC_003291(11)
NODE_30_length_11860_cov_30.694792	Questionable	13	226-9582	Lister_2389_NC_003291(11)
*Bacillus bombysepticus* 2D3-3				
NODE_1_length_896591_cov_44.627984	Incomplete	31	229-21688	Bacill_vB_BceS_MY192_NC_048633(8)
NODE_3_length_454515_cov_31.451986	Intact	55	17783-64029	Bacill_phBC6A51_NC_004820(31)
NODE_6_length_333245_cov_46.434435	Incomplete	29	262297-295042	Brevib_Abouo_NC_029029(3)
NODE_8_length_279631_cov_38.733918	Intact	41	136571-191583	Geobac_GBSV1_NC_008376(8)
NODE_10_length_258922_cov_35.575023	Incomplete	24	919-25714	Bacill_phi4J1_NC_029008(3)
NODE_14_length_171494_cov_28.655482	Incomplete	39	133494-169236	Bacill_phBC6A52_NC_004821(12)
NODE_30_length_11867_cov_43.929131	Questionable	13	250-9588	Lister_2389_NC_003291(11)
NODE_31_length_11860_cov_26.021222	Questionable	13	226-9582	Lister_2389_NC_003291(11)
*Bacillus altitudinis* 2R-9				
NODE_3_length_375146_cov_59.925193	Incomplete	0	334511-362821	Brevib_Jimmer1_NC_029104(7)

### Mining for antibiotic-resistant and virulence genes

**Fig 8:**
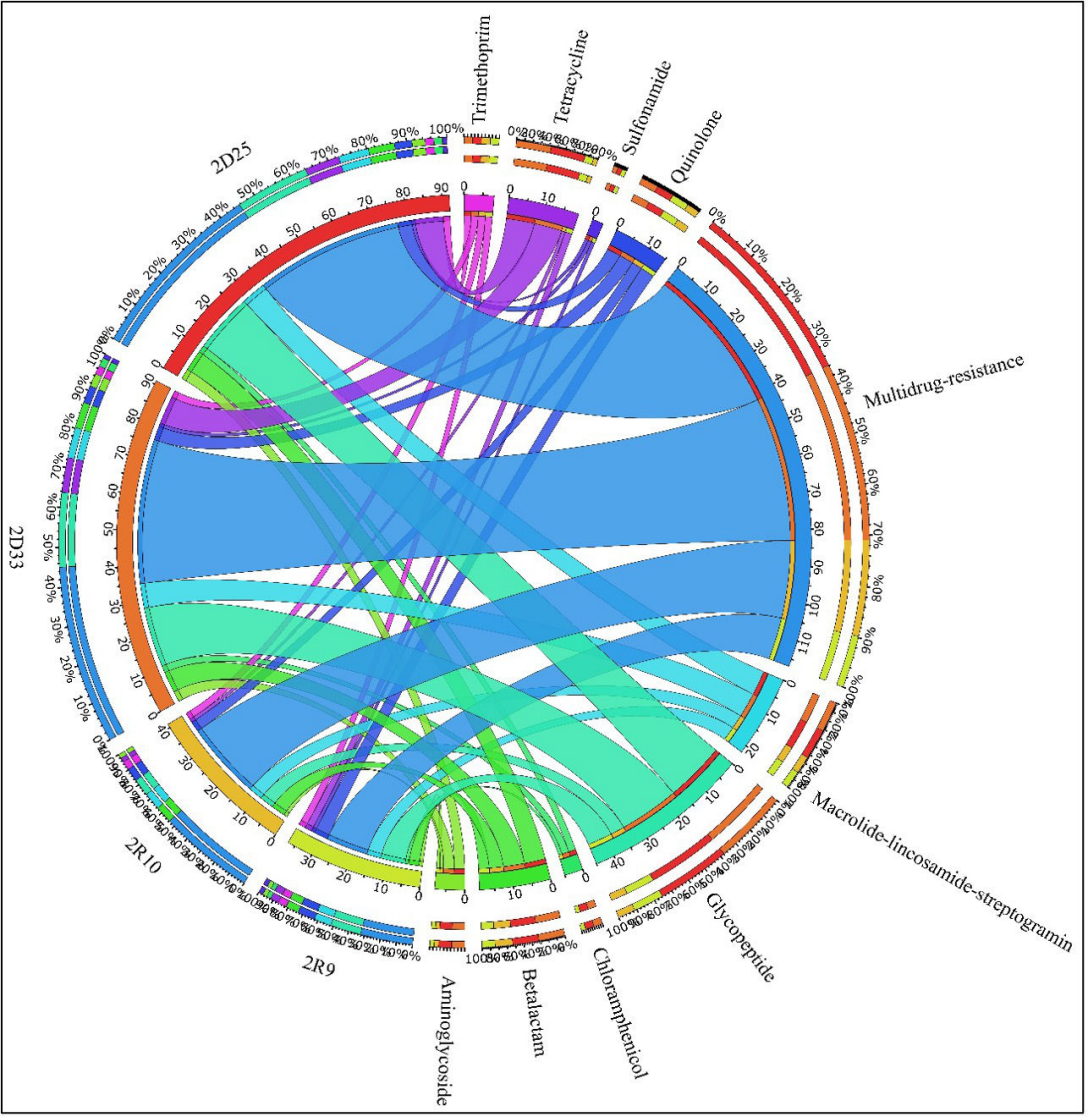
Abundance of antibiotic resistance genes in HPC isolates based on DeepARG.

**Fig 9:**
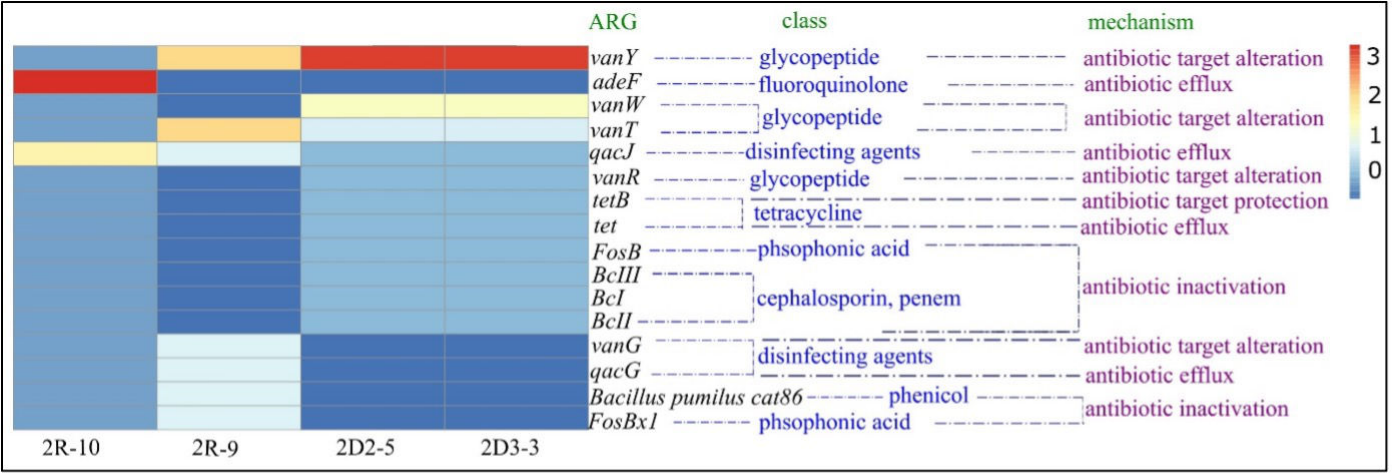
ARGs predicted from by RGI in CARD database. The heatmap represents the number of the ARG identified by the CARD database under a strict level. Light blue shows the lowest number of ARG identified, while red shows the highest number identified in each isolate. The class and mechanisms of action of each identified ARGs are presented along with the heatmap.

The two raw water isolates revealed 36 and 41 genes involved with antibiotic resistance, 47 and 92 genes were identified in the two treated water isolates, whereas the two isolates from distribution water revealed 92 and 91 different ARGs based on DeepARG. These ARGs belonged to 10 groups which include multidrug-resistant gene groups, macrolide-lincosamide-streptogramin, glycopeptide, tetracycline, aminoglycoside, sulfonamide, quinolone, β-lactam, chloramphenicol, and trimethoprim. Figures 8 and 9 show the abundance of the antibiotic resistance genes identified in relation to the antibiotics for which these genes encode resistance from DeepARG and CARD, respectively. The multidrug-resistant gene group contained the most identified genes for all four isolates. This was followed by glycopeptide and tetracycline. The number of identified resistance genes increased from the raw water isolates to the drinking water. The number of genes identified in distribution water (2D2-5 and 2D3-3) increased to more than double that identified in the raw water isolates (2R-9 and 2R-10).

DeepARG also predicted up to 103 possible new ARGs in the raw water, 162 in the treated water, and the distribution water. Based on this prediction, three extra groups were added includes bacitracin, fosfomycin, and polymyxin. It needs to be noted that this prediction had less than 30% amino acid similarity with the database.

**Fig 10:**
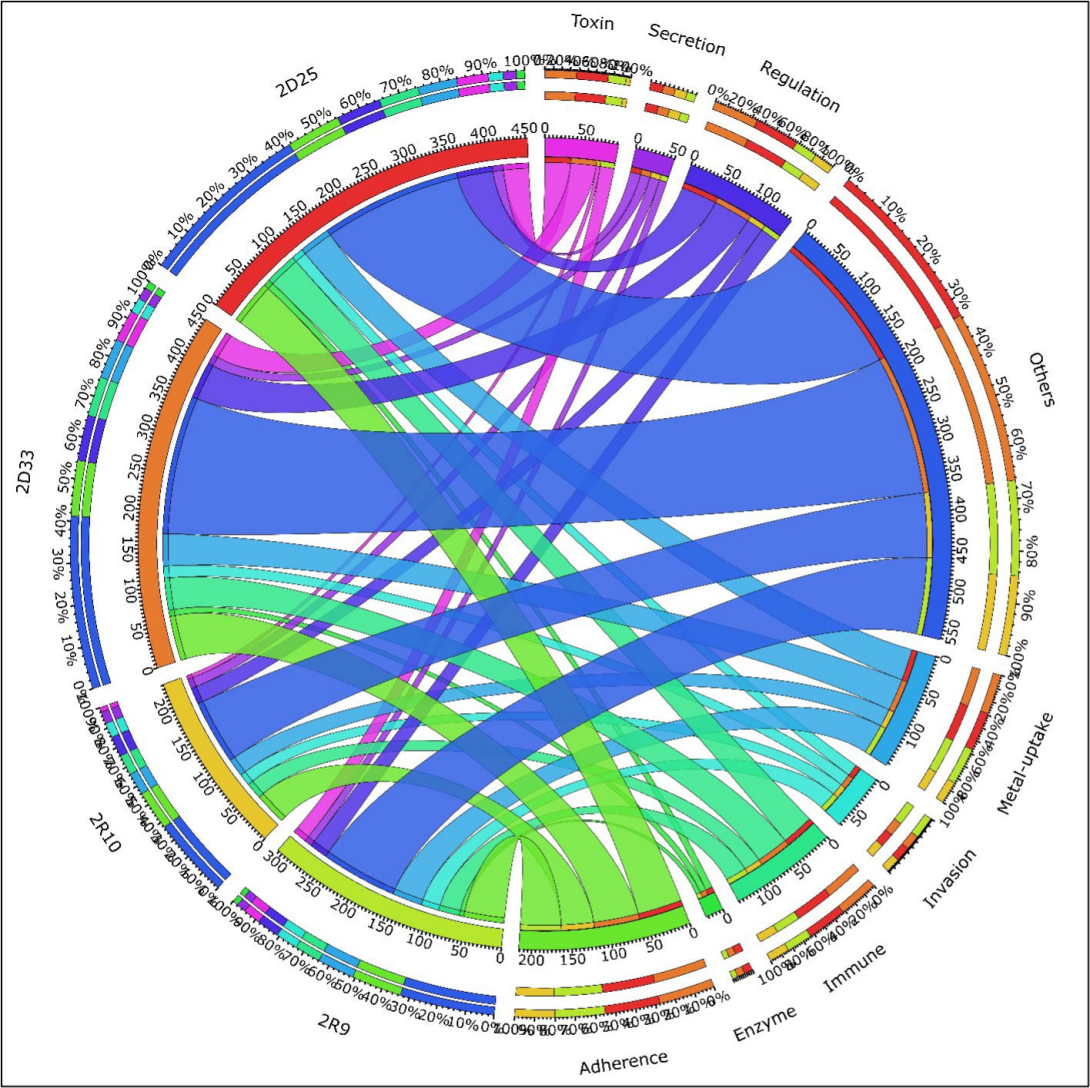
Abundance of virulence genes from WGS analysis based on DeepARG.

Virulence genes were also identified and associated with specific characteristics such as immune, adherence, enzyme production, invasion, metal uptake, regulation, secretion, toxin, etc. Illustrated in Fig. 10 from DeepARG from the virulence factor database, it is clear that virulence genes identified under the group “other” were the most prevalent by reaching 102 genes in the raw water and 187 genes in the treated water as well as the distribution water. Genes in the adherence group ranged from 45 in the raw water to 61 in the drinking water. The number of genes in the immune group ranged from 23 in the raw water to 44 in the drinking water. The regulation group had 20 genes from the raw water and increased to 50 in the drinking water. The metal uptake group had 25 genes in raw water and increased to 43 in the drinking water. Once again, it is observable that with most virulence genes, there was an increase from the raw water to the drinking water except for genes encoding the invasion group, which were mostly present in the raw water.

## DISCUSSION

Antibiotic residues in water samples have been identified as contaminants that affect water quality and contribute to the prevalence of antibiotic resistance (44). Low levels of antibiotics are found in sewage water, soils, and aquatic habitats due to contamination from human activities, including natural production. Concerns arise regarding potentially transferring these chemical substances into drinking water through treatment processes. Our study confirmed these concerns, as ciprofloxacin, neomycin, benzyl-penicillin (penicillin G), penicillin, and streptomycin were detected in the drinking water (Table 1). Other studies have also detected antibiotics, such as erythromycin and sulfamethoxazole, in water samples (45). Sub-therapeutic doses of antibiotics can activate bacterial repair systems, leading to increased gene transfer responsible for antimicrobial resistance (46, 47).

Potentially pathogenic heterotrophic bacteria pose a threat to humans, particularly those with underlying infections or compromised immune systems, and their antibiotic resistance exacerbates the situation. Hemolytic bacteria, which break down erythrocytes to access iron for growth, play a significant role in pathogenesis (48). Our study revealed a high proportion of hemolytic isolates (76.5%–87%) surpassing previous reports (25, 49), indicating the potential pathogenicity of most heterotrophic bacteria. These isolates also exhibited enzyme secretion, including proteinase, lecithinase, lipase, and DNase, facilitating penetration and access to host cells (50, 51). These findings indicate invasiveness and the potential to cause infection, highlighting the pathogenic characteristics of heterotrophic strains and the associated health risks, especially for immunocompromised individuals and vulnerable groups.

Nine isolates from NWC and NWG drinking water exhibited the secretion of all tested enzymes. These isolates include bacteria such as *Bacillus* spp., *Bacillus paramycoides*, *Arthrobacter oryzae*, *Arthrobacter* spp., *Bacillus sonorensis*, *Shewanella* spp., *Bacillus licheniformis*, *Deefgea rivuli*, and *Aeromonas salmonicida*. Previous studies have identified some of these bacteria as opportunistic pathogens frequently found in drinking water (52). *Bacillus* spp. are often detected in drinking water, even with appropriate treatment and disinfection procedures (53). Although *Bacillus* spp. are not commonly associated with waterborne pathogens, they should not be underestimated regarding potential pathogenicity (54). *Aeromonas salmonicida* has been reported in human infections and can colonize various water sources (55). The presence of these potential pathogens further emphasizes the need for preventive measures.

Resistance against β-lactams (ampicillin, cephalothin, and penicillin) and trimethoprim was observed in heterotrophic strains at both treatment facilities, which may correlate with their frequent use in clinical practice (56). High resistance patterns indicate the widespread consumption of these antibiotics and the need for responsible antibiotic use. Our study’s MARI values indicated a potential origin from high-risk sources with significant antibiotic usage (57). The increased resistance from raw to distributed water suggests the selection of bacteria with survival and resistance traits during treatment processes (15).

Whole genome sequence analysis revealed the presence of ARGs in the isolates, including multidrug-resistant gene groups and genes associated with various antibiotics (58). Genome islands were identified, which can carry ARGs and facilitate their transfer through horizontal gene transfer (59). The analysis also identified phages, which can play a role in antimicrobial resistance and gene transfer (60, 61). Comparative genomics provided insights into the isolates’ genomic features and evolutionary relationships (62, 63).

Our study highlights the presence of antibiotic residues in water samples and their potential impact on antibiotic resistance. Potentially pathogenic heterotrophic bacteria, including hemolytic strains, exhibited enzyme secretion, indicating their pathogenic characteristics. Certain isolates from drinking water showed the secretion of all tested enzymes, indicating their potential pathogenicity. Resistance against β-lactams and trimethoprim was observed, reflecting consumption trends and the need for responsible antibiotic use. Whole genome sequence analysis revealed the presence of ARGs, phages, and genome islands, emphasizing the role of genetic factors in antimicrobial resistance. These findings underscore the importance of monitoring and managing water quality to mitigate health risks associated with antibiotic-resistant heterotrophic strains.

### Conclusions

Although heterotrophic bacteria are often regarded as not posing any health risks in drinking water, the findings of this study revealed that more than 80% of the heterotrophic bacterial isolates showed potential pathogenic properties. This indicates that these enzymes may allow them to be invasive and enter host cells, which is the first step toward infection. Elevated levels of resistance to β-lactams and trimethoprim were documented. The mean percentage of antibiotic resistance observed among the isolates obtained from NWC and NWG varied from 47% to 100%, indicating a significant prevalence. The second trend that has been noted pertains to the escalation in resistance levels of antibiotics from raw water to drinking water at NWC and NWG. The treatment procedures appear to favor the proliferation of bacteria with higher resistance. The findings indicate that the isolates under investigation have a high-risk source of contamination where antibiotics are utilized, as evidenced by the MARI values surpassing the threshold of 0.2. The detection of antibiotic residues in the water samples highlights the need for effective monitoring and management strategies. Sequence analysis of four representative species from NWC showed that these bacteria contain various antibiotic-resistant genes and virulence factors, mainly of the multidrug-resistant gene family, which correlated with the phenotypic patterns observed. The WGS data reveal a discernible rise in antibiotic-resistant and virulence genes from the untreated water source to the potable water, suggesting that water treatment may significantly influence the selection, dispersion, and prevalence of antibiotic-resistant bacteria.

In addition, identifying antibiotic resistance genes and mobile genetic elements through genome sequencing analysis provides insights into the genetic basis of resistance and the potential for horizontal gene transfer. These findings underscore the importance of continuous surveillance and the development of strategies to mitigate the risks associated with pathogenic heterotrophic bacteria and antibiotic resistance in drinking water systems.

Finally, policymakers and public health authorities should intensify monitoring of antibiotic resistance in drinking water, revising treatment strategies to minimize the proliferation of resistant strains, strengthen regulations, invest in advanced treatment technologies, and promote responsible antibiotic use. Furthermore, educating healthcare providers and the public, supporting research on resistance mechanisms, integrating genomic surveillance, establishing emergency response plans, and fostering international collaboration are key components to mitigate the risks associated with antibiotic resistance in drinking water systems.

## Data Availability

The whole-genome shotgun project for isolates 2D2-5, 2D3-3, 2R9, and 2R10 has been deposited at DDBJ/ENA/GenBank under accession numbers JASDAQ000000000, JASDAR000000000, JASCXE000000000, and JASCXF000000000, respectively.

## SUPPLEMENTAL MATERIAL

Supplemental tables spectrum.03359-23-s0001.xlsx Tables S1 to S9.

ASM does not own the copyrights to Supplemental Material that may be linked to, or accessed through, an article. The authors have granted ASM a non-exclusive, world-wide license to publish the Supplemental Material files. Please contact the corresponding author directly for reuse.
